# Autoantibodies in Neuropsychiatric Disorders

**DOI:** 10.3390/antib5020009

**Published:** 2016-04-21

**Authors:** Carolin Hoffmann, Shenghua Zong, Marina Mané-Damas, Peter Molenaar, Mario Losen, Pilar Martinez-Martinez

**Affiliations:** Division Neurosciences, School for Mental Health and Neurosciences, Maastricht University, 6200 MD Maastricht, The Netherlands; c.hoffmann@maastrichtuniversity.nl (C.H.); s.zong@maastrichtuniversity.nl (S.Z.); m.damas@maastrichtuniversity.nl (M.M.-D.); p.molenaar@maastrichtuniversity.nl (P.M.); m.losen@maastrichtuniversity.nl (M.L.)

**Keywords:** autoantibody, neuronal surface antigens, neuropsychiatric disorders, effector mechanisms, immunoglobulin, blood brain barrier, NMDA-R, VGKC complex, AMPA-R, GABA-R, DR

## Abstract

Little is known about the etiology of neuropsychiatric disorders. The identification of autoantibodies targeting the *N*-methyl-d-aspartate receptor (NMDA-R), which causes neurological and psychiatric symptoms, has reinvigorated the hypothesis that other patient subgroups may also suffer from an underlying autoimmune condition. In recent years, a wide range of neuropsychiatric diseases and autoantibodies targeting ion-channels or neuronal receptors including NMDA-R, voltage gated potassium channel complex (VGKC complex), α-amino-3-hydroxy-5-methyl-4-isoxazolepropionic acid receptor (AMPA-R), γ-aminobutyric acid receptor (GABA-R) and dopamine receptor (DR) were studied and conflicting reports have been published regarding the seroprevalence of these autoantibodies. A clear causative role of autoantibodies on psychiatric symptoms has as yet only been shown for the NMDA-R. Several other autoantibodies have been related to the presence of certain symptoms and antibody effector mechanisms have been proposed. However, extensive clinical studies with large multicenter efforts to standardize diagnostic procedures for autoimmune etiology and animal studies are needed to confirm the pathogenicity of these autoantibodies. In this review, we discuss the current knowledge of neuronal autoantibodies in the major neuropsychiatric disorders: psychotic, major depression, autism spectrum, obsessive-compulsive and attention-deficit/hyperactivity disorders.

## 1. Introduction

Schizophrenia, major depressive disorder (MDD), and bipolar disorder (BD) were classically seen as psychiatric disorders or mental illness, which classifies a disturbance of the “mind”. This classification developed within the paradigm of dualism in which mind and body are separated [1]. As such, psychiatric disorders were distinguished from the neurological diseases which have a demonstrable pathology. With today’s understanding of both fields, the distinction is only based on symptomatology because both classifications have detectable biological causes. Due to these developments in psychiatry, the subspecialty of neuropsychiatry is growing. Accordingly, in neuropsychiatric disorders both psychiatric symptoms (affecting emotions, thoughts and behaviors) and neurological symptoms (movement disorders, epileptic seizures and cognitive impairment) can be identified. The occurrence of one of these symptoms does not necessarily yet lead to the diagnosis of a certain psychiatric or neurological disease; an isolated epileptic attack is not epilepsy and an isolated psychotic episode is not schizophrenia. Only when symptoms are persisting over a certain time, this diagnosis will be made. Notwithstanding that the knowledge of biological psychiatry is advancing, the diagnosis of these syndromes is still based on behavioral phenotypes following the classification from the Diagnostic and Statistical Manual of Mental Disorders (DSM, currently version 5) and the International Statistical Classification of Diseases and Related Health Problems (ICD, currently version 10). These diagnoses are still not trivial because the rating of psychiatric symptoms is challenging (although important efforts have been made to objectivize the diagnosis [2]) and many neuropsychiatric disorders have overlapping symptoms. Consequently, classification guidelines keep changing during the years.

The etiology of neuropsychiatric disorders is very diverse and still poorly understood but a set of biological changes and risk factors have been identified for the different diagnoses. Some of these disease mechanisms are overlapping between different neuropsychiatric disorders, with the major biological changes being the alteration of synaptic transmission, including hypofunction of dopamine receptor (DR) and *N*-methyl-d-aspartate receptor (NMDA-R) and, also, dysfunction of the voltage gated potassium channel (VGKC) [3,4,5]. Inflammation is associated with neuropsychiatric etiology, probably caused by infections or autoimmune diseases. In recent years, the discovery of certain autoantibodies targeting the central nervous system (CNS) such as α-amino-3-hydroxy-5-methyl-4-isoxazolepropionic acid receptor (AMPA-R), γ-aminobutyric acid (GABA-R), and the metabotropic glutamate receptors (mGluR) could be an important breakthrough in neuropsychiatry. Autoantibodies targeting mainly neuronal membrane proteins have now been revealed to potentially alter memory, behavior, and cognition or cause psychosis, seizures, and abnormal movements [6,7]. These autoimmune encephalopathies also have an implication in psychiatry as some of these autoantibodies (such as anti-NMDA-R) are seen in patients with psychotic symptoms that have not previously been considered to have an autoimmune origin [8]. Thus, psychotic disorders and autoimmune encephalitis have overlapping symptoms. During the last years, effort has been made to better understand the autoimmune mechanisms that can induce neuropsychiatric disorders. Here we review antibody mediated autoimmunity against neuronal (membrane) proteins in five major neuropsychiatric disorders: psychotic, major depressive (MDD), autism spectrum (ASD), obsessive-compulsive (OCD) and attention-deficit/hyperactivity (ADHD) disorder. Table 1 summarizes the characteristics, prevalence and etiology of these disorders.

## 2. Indications for Autoimmune Mechanisms in Neuropsychiatric Disorders

Genetic studies of large sample sizes have revealed several gene variants that increase the risk of neuropsychiatric disorders, including genes encoding for neurotransmitter receptors and ion channels. In the Psychiatric Genomics Consortium, single nucleotide polymorphisms (SNPs) in two l-type voltage-gated calcium channel subunits, voltage-gated calcium channel subunit alpha1 C (CACNA1C) and calcium voltage-gated channel auxiliary subunit beta2 (CACNB2), were identified as common risk factor among all studied diagnosis including ASD, ADHD, BD, MDD and schizophrenia [35].

In addition, variants from the human leukocyte antigen (HLA) region (major histocompatibility complex; MHC molecules) which are involved in antigen presentation have not only been associated with the risk of developing autoimmune diseases, but also with the risk of developing several neuropsychiatric disorders. A C4B null allele, a deficient form of the HLA C4B gene (no C4B protein produced), was reported to be more frequent in ASD, ADHD and dyslexia [12]. Another locus called the HLA DRB1 was implicated in schizophrenia and ASD as well as autoimmunity [36,37]. Taken together these findings suggest that neuroinflammation and autoimmunity may play a role in neuropsychiatric disorders [13].

Autoantibodies in neuropsychiatric disorders cause mainly a loss rather than a changed pattern of channel activity, possibly associated with neuroinflammation and neurodegeneration [38,39,40]. To demonstrate autoimmune pathogenicity according to Witebsky’s postulates, four conditions have to be met: (1) the autoantibody must be present with the clinical manifestation and detectable in the blood and/or affected tissue; (2) autoantibodies should target a receptor, ion channel, or other protein expressed on the membrane surface; (3) antibody transfer can replicate the disease in an animal experimental model or in humans (maternal transfer); and (4) elimination or suppression of the autoimmune response by therapy can prevent disease progression or improves the clinical manifestations.

## 3. Ion Channels and Receptor Functions

Autoantibodies in neuropsychiatric disorders commonly target neuronal ion channels or associated proteins. For an in-depth understanding how autoantibodies against these molecules cause disease, it is necessary to comprehend the functions of neuronal ion channels and receptors, which largely determine the inter-neuronal communication and properties of neurons. These channels are facilitating the depolarization, hyperpolarization and also repolarization of neurons and thus are essential to the signal transmission and functioning of the brain [41]. The basis of the transmission of electric currents is the membrane potential, which is the difference in electrical charge between the inside and outside of neurons. This difference is produced by ion pumps that create high extracellular Na^+^, Cl^−^ and Ca^2+^ concentrations and high K^+^ inside of the neuron. During an action potential, depolarization induces rapid influx of Na^+^ followed by slightly slower opening of K^+^ channels that induce repolarization and thereby enable a relatively fast subsequent activation of the neuron. Within the nervous system, different neurons possess a unique mixture of a wide variety of ion channels, which characterize their electrophysiological properties. The different types of synapses are largely defined by the neurotransmitter that is used for signal transduction, which are acetylcholine (ACh), noradrenaline (NA), dopamine, glutamate (Glu), serotonin (5HT), γ-aminobutyric acid (GABA, inhibitory), glycine (Gly, inhibitory), nitric oxide and a series of peptide neurotransmitters including endorphin.

Neurotransmitters can activate these receptors by either inducing a direct opening of an ion channel (ionotropic receptor) or altering the concentration of intracellular metabolites via GTP binding proteins (metabotropic receptors). Due to their mode of action, ionotropic receptors, such as the NMDA-R, promote a rapid signal transduction and are responsible for the majority of neuronal communication in the CNS and peripheral nervous system (PNS). On the other hand, metabotropic receptors, including the metabotropic Glu receptors (mGluRs) and the D2 dopamine receptor (D2DR), act via second messengers and therefore have a slower effect but also longer duration of action and can lead to long-term changes such as synaptogenesis.

Conceptually, it makes sense that binding of autoantibodies to these receptors which have the potency to interfere with the action of these fundamental signaling processes can induce severe neurological and psychiatric symptoms. This is further supported by the fact that substances with an inhibitory effect on neurotransmitter receptors, such as ketamine, acting on NMDA-R or lysergic acid diethylamide on 5HT receptors (5HT-R) are potent hallucinogens.

## 4. The Role of Blood-Brain Barrier Integrity on Autoantibody Effects

Despite tight immune surveillance of the CNS, antibodies cross in low numbers through the blood-brain barrier (BBB). Once they reach the cerebrospinal fluid (CSF), the turn-over is about four times per day. This dynamic equilibrium results in immunoglobulin G (IgG) levels in the brain that amount to about 1% of the plasma levels, and in about 10% of the total protein in the CSF [38,42,43]. In autoimmune encephalitis it is known that several autoantibodies cross the BBB and can be detected in the CSF. However, the mechanism of how antibodies cross to the CSF is not very well understood. The permeability of the BBB is altered upon damage and inflammation of the brain. Additionally, an impaired function of apolipoprotein E (ApoE) has been shown to reduce the barrier function of tight junctions [44]. This knowledge was used to study whether an impaired BBB would change the effect of peripherally administered human NMDA-R antibodies in a mouse model. Hammer *et al.* claim that only in ApoE knock out mice but not in wild type mice, human NMDA-R antibodies cause psychosis-related behavioral perturbation [45]. The same study also relates the effects of the autoantibodies to the patients’ history of birth complications or neurotrauma indicating possible BBB insufficiency. This hypothesis is further supported by the findings that increased prevalence of psychiatric comorbidity in diseases is associated with BBB dysfunction, including systemic lupus erythematous (SLE) [46,47,48], stroke [49,50,51,52], epilepsy [53,54] and autoimmune encephalitis [45,55]. An increased albumin ratio in CSF to serum in patients with MDD and schizophrenia further suggests increased BBB permeability [56]. On the other hand, circulating B cells cross the BBB during normal immune surveillance [57] which might include antibody producing cells. CD138+ plasma cells, were found in post-mortem and biopsy tissue of NMDA-R encephalitis patients [58]. Intrathecal antibody production was also described in Sydenham chorea (SC) patients with anti-lysoganglioside GM1-specific IgG [56] and in a case with autoantibodies against the GluN1 subunit (also known as NR1) of the NMDA-R where the patient did not respond to plasmapheresis treatment, while plasma antibody levels dropped but CSF levels remained high [59]. Some groups report that in patients with encephalitis autoantibodies against NMDA-R, AMPA-R, metabotropic or B class of the GABA-R (GABAB-R), dipeptidyl-peptidase-like protein-6 (DPPX), mGluR1 or mGluR5 can always be found in the CSF whereas other autoantibodies, such as autoantibodies to leucine-rich glioma inactivated-1 (LGI1), to contactin associated protein-2 (CASPR2), to glycine receptor (GlyR) and to the ionotropic or A class of the GABA-R (GABAA-R) may, in rare instances, be identified only in serum [7]. If no autoantibodies can be detected in the CSF, it is unclear how they can have central effects and thus if they are pathogenic. However, if the autoantibodies are present but immuno-absorbed by the antigen in the brain, they might not be detectable in the CSF [60].

In addition, T cells might have a role in BBB integrity and thus antibody penetration. Recently, Dileepan and colleagues described that T-helper 17 cells activation caused by group A *Streptococcus* infection disrupt the integrity of the BBB, and facilitate circulating autoantibodies to enter the brain [61].

## 5. Transfer of Autoantibodies via the Placenta

Transfer of maternal IgG antibodies to the fetus is a protective mechanism during the period in which the infant has an undeveloped humoral immune response [62]. IgG antibodies are the only Ig isotype that crosses the placenta and they do so via neonatal Fc receptors (FcRn) on syncytiotrophoblast cells. The amount of IgGs passing to the fetus is altered dependent on e.g. maternal levels of specific antibodies, period of gestation, placental integrity and type of antigen. If the mother has IgG autoantibodies in the blood, these will also be transferred to the neonate where they can induce pathogenic effects. Additionally, it has been seen in a rat model that in the fetus, the IgG penetration to the brain is higher than in the adult [63], indicating that these autoantibodies might reach and bind neuronal receptors in the fetus. Such an example is autoantibodies targeting the acetylcholine receptor (AChR) located at the neuromuscular junction (NMJ) which is composed of five subunits. Receptors are either of the embryonic form, composed of α1, β1, γ and δ subunits, or of the adult form composed of α1, β1, δ and ε subunits. Mothers carrying autoantibodies specifically against the gamma subunit (AChRγ) are frequently asymptomatic [64,65]. Maternal antibodies of this sort can impair skeletal muscle development and cause fixed joint contractures and other deformities called arthrogryposis multiplex congenita. In other neurodevelopmental disorders such as autism [66,67,68] and dyslexia [69], a role of maternal autoantibodies has been suggested (see section on ASD later). In SLE, a pathogenic transfer of maternal antibodies has been described [70] and maternal antibodies have been hypothesized to cause long-term cognitive changes since children born to mothers with SLE display high incidence of learning disorders [71,72,73]. In a mouse model with high maternal autoantibody levels targeting double stranded DNA (dsDNA) and cross-reacting with GluN2a/2b subunits of NMDA-R, cognitive impairments in adult offspring have been detected due to histological abnormalities in fetal brain [74]. Taken together, these studies suggest that *in utero* exposure to neurotoxic/inflammatory autoantibodies generates developmental abnormalities with long-term consequences. In some cases, the effects of neonatal autoantibody exposure might only present later in life and potentially only with certain environmental exposures which makes it very difficult to study these disease mechanisms. In case that the presence of maternal autoantibodies can be detected, these complications are treatable during pregnancy with intravenous IgG (IVIg) that competes with the endogenous autoantibodies, saturate FcRn and increase IgG turnover [75].

## 6. Autoantibody Effector Mechanisms

Autoimmune diseases are induced by complex immune dysfunctions of T-cells, B-cells and other immune cells, but can be simply classified as T-cell or antibody mediated. It is still largely unknown which mechanisms are involved in autoimmune neuropsychiatric disorders, however, most studies point towards an antibody mediated pathology. We will therefore focus here on the IgG antibody mediated disease mechanisms, which can be summarized as follows:

(a) Complement deposition and inflammation is a common mechanism in autoimmune diseases. The complement system is part of the innate immune system and can be activated by antibody-antigen complexes, which leads to the activation of complement proteins amplifying its effector mechanisms [39,76]. Effects of complement are (i) opsonization and engulfment by phagocytes with receptors for complement; (ii) chemo-attraction and activation of phagocytes and (iii) formation of the so called membrane attack complex in cell membranes leading to lysis, extensive tissue damage and loss of tissue architecture, including receptors and ion channels.

For example, complement activation is important in Rasmussen's encephalitis, where autoantibodies anti-GluR3 subunit of the AMPA-R have been detected [77]. Peripherally, anti-AChR autoantibodies (of IgG1 and IgG3 isotype) from myasthenia gravis (MG) patients [78,79] activate the classical complement pathway. This causes complement deposition in the NMJ, where the antigen is located, resulting in a morphological damage and loss of the AChR in the post-synaptic membrane [39].

(b) Stimulation or inhibition of receptor function can be induced upon binding of the autoantibody without further activation of the immune system. Examples for this mechanism are autoantibodies targeting the folate receptor (FR) which have a very high binding affinity and thereby block binding and uptake of folic acid [80]. This inhibits the transport of folic acid into the CSF and causes cerebral folate deficiency leading to infantile-onset neuropsychiatric symptoms including psychomotor retardation, cerebellar ataxia, dyskinesias and in some cases, seizures. The autoantibodies found in SC patients alter the D2DR function by reducing adenylate cyclase levels in a comparable level to the inhibitory effect by dopamine [81]. By targeting the receptor the autoantibodies can also interfere with the intracellular signaling pathways activated by the calcium/calmodulin-dependent (CaM) kinase II, an enzyme involved in cognition and neurotransmitter synthesis and release [82,83,84]. The activation of this enzyme has been correlated with an increase of dopamine release in the brain [85].

(c) Antigen internalization (or antigenic modulation) is a mechanism in which binding of autoantibodies induces an internalization and commonly degradation of the antigen. The two arms of the antibody can bind each separately to an antigen leading to clustering or cross-linking of the antigens in the membrane. Antibodies against the NMDA-R are thought to cause cross-linking and selective internalization of receptors as shown in cultured neurons [86,87]. Reduction in DR levels has also been observed in the presence of SC patient autoantibodies [81]. In the PNS, specifically in MG, anti-AChR autoantibodies accelerate the internalization of the receptor [88,89], a mechanism that can be blocked by overexpression of the AChR anchoring protein, rapsyn, in an experimental passive transfer MG model, showing the important role of anchoring proteins in the resistance to the autoantibody attack [90].

(d) Loss or block of receptor associated proteins can also significantly alter the function of ion channels. One of the known antigens associated with the VGKC is LGI1 [91,92]. LGI1 autoantibodies can cause a disruption of the ligand-receptor interaction of LGI1 with scaffolding proteins ADAM22 or ADAM23, which is interfering with the trans-synaptic complex that includes presynaptic Kv1.1 potassium channels and post-synaptic AMPA-R [93,94]. It has also been observed in the PNS that the AChR internalization and complement damage produces a loss of scaffolding proteins associated to the receptor like muscle-specific kinase (MuSK), rapsyn, docking protein 7 (Dok-7), LDL Receptor Related Protein 4 (Lrp4) or agrin, altering the endplate organization and, in some cases, aggravating the symptoms and delaying the repairing mechanisms [39].

## 7. Relevance of Intracellular Antigens as Target of Autoimmunity

Considering the pathologic mechanisms described above, autoantibodies are unlikely to be pathogenic if they target intracellular antigens such as Hu, Yo or Ri, as commonly seen in paraneoplastic syndromes [95]. Instead, diseases with intracellular antigens are thought to be T-cell mediated [96] and are not within the scope of this review. The pathogenicity of a few autoantibodies targeting intracellular antigens e.g. amphiphysin, glutamic acid decarboxylase (GAD), ribosome P proteins (Rib-P) and anti-dsDNA, is still controversial. Amphiphysin is a synaptic vesicle protein which might be exposed to autoantibodies in the membrane during synaptic vesicle uptake [7] and has been described as an antigen affected by autoantibodies in Stiff Person syndrome. Amphiphysin autoantibodies induced structural disorganization in GABAergic synapses and changed presynaptic vesicle pools [97]. In addition, autoantibodies to GAD, the enzyme synthesizing the inhibitory neurotransmitter GABA, are related to many neurological disorders e.g. Stiff Person syndrome, cerebellar ataxia, limbic encephalitis (LE), epilepsy and oculomotor dysfunction [98]. Gresa-Arribas and collaborators observed that anti-GAD autoantibodies are not internalized by neuronal cell cultures, indicating that the antibodies are unlikely to interact with GAD on live neurons [99]. Epitope specificity overlaps between different syndromes with GAD autoantibodies and thus cannot explain the differences in symptoms [98,99].

If the anti-GAD autoantibody is the causative factor, other antibody properties or interacting factors such as environmental factors must also play a role. The study of these autoantibodies is of clinical relevance as potential diagnostic marker, because GAD autoantibodies in classical paraneoplastic syndrome are indicative for identification of tumors and often coincide with other autoantibodies that target neuronal antigens such as GABA-R [100,101] and GlyR [102,103]. Studies in rats showed that injection of IgG from patients with GAD autoantibodies and neurological symptoms lead to motor dysfunction and impaired NMDA-R signaling but not when injecting IgG from GAD positive diabetes patients without neurological presentation [104], which was interpreted as pathogenic role of autoantibodies related to neurological symptoms. Other authors claim that these changes were evoked by accompanying other autoantibodies [101]. Interestingly, the repertoire of antibodies to different immunodominant regions in the GAD antigen is wider in the CNS than systemically [99].

Autoantibodies against Rib-P have been proposed to be involved in the neuropathogenicity in psychiatric SLE, e.g., anti-Rib-P autoantibody titers were correlated to depression in the onset of SLE [105]. A murine model illustrates the ability of anti-Rib-P autoantibodies to induce depressive-like symptoms [106,107]. Moreover, Matus *et al.* showed that these antibodies could cross-react with a novel neuronal surface protein causing Ca^2+^ influx and apoptosis. However, there exists in the literature some controversy in the association of anti-Rib-P with CNS involvement and neuropsychiatric manifestations in SLE [108,109], which may be due to the great variation in detection assays concerning the purity of the anti-Rib-P autoantibodies, the use of synthetic peptides, or parts/complete antigen as well as the carrier proteins used. Anti-dsDNA autoantibodies have been proposed to cross-react with the GluN2 subunit of the NMDA-R and are responsible for excitatory, non-inflammatory cell death and altered neuronal function [110].

In Table 2 the presence of autoantibodies in neuropsychiatric diseases targeting membrane and intracellular proteins is summarized; the latter will be discussed in the following section. Table 3 gives an overview of autoantibodies targeting membrane proteins and intracellular antigens only in psychiatric disorders

## 8. Autoimmune Encephalitis

Encephalitis is an inflammation of the brain characterized by memory alterations, behavioral and cognitive changes and seizures, where immune mediated mechanisms have been related [111]. Paraneoplastic events are very common in encephalitis patients and antibodies to intracellular onconeuronal antigens and cytotoxicity mechanisms have been described [112]. In Table 2, the autoantibodies involved in neurological diseases with psychiatric symptoms are summarized.

A few years ago, NMDA-R autoantibodies were described for the first time in a group of encephalitis patients with ovarian teratoma with the peculiar fact that they suffered psychotic symptoms. Autoantibodies were identified in serum and CSF using a cell based assay (CBA) (HEK293 cells expressing single subunits or dimers of the GluN1 and GluN2a/2b) and rat brain immunohistochemistry (IHC) [123]. Importantly, about 80% of these patients had full or substantial recovery after treatment with immunotherapy and removal of the tumor if present, which indicates that the antibody is an important, if not, the only cause of symptoms. This further implies that subgroups of patients with neuropsychiatric disorders are treatable with immunotherapy. These findings were confirmed in other encephalitis cohorts [122,132] and in individual patients [133] with a case example of a young lethargic encephalitis patient, who had high NMDA-R autoantibodies (higher in CSF than in serum) and responded well to immunosuppressive therapy [134]. For NMDA-R autoantibodies, higher sensitivity and specificity was found in studies using CSF [116,118,134]. In contrast, using a fluorescent immunoprecipitation assay anti-GluN1, IgG autoantibodies were found in higher levels in serum than in CSF in 10% of cases [114]. The GluN1 subunit of the receptor was identified as the main antigenic epitope in the classic full spectrum NMDA-R encephalitis cases [117,135], specifically a small region in the amino terminal domain [136]. Upon binding, the autoantibodies are thought to cause cross-linking and selective internalization of NMDA-R as shown in cultured neurons [86]. It has also been proposed that in the extrasynaptic compartment, autoantibodies significantly reduce the surface diffusion of NMDA-R, likely facilitating their internalization and degradation [87]. An important contribution to verify the effect of these autoantibodies was recently given by Planagumà and colleagues who showed that passive transfer of NMDA-R autoantibodies by continuous intraventricular infusion of CSF from patients with NMDA-R encephalitis causes memory and behavioral deficits in mice. The passive transfer model was also able to reproduce the downregulation of total and synaptic NMDA-R density observed in the disease in humans. After discontinuing the patient CSF infusion, the NMDA-R clusters in the hippocampus, and the total NMDA-R protein amount was recovered gradually, supporting the reversible effect of these autoantibodies [137,138].

Patients with autoantibodies against the VGKC complex have been reported in many neurological disorders (including LE, epilepsy, neuromyotonia) as immunotherapy-responsive [139]. Radioimmunoassay (RIA) and IHC techniques were used to test VGKC reactivity in serum and CSF. Higher percentages (17%–26%) of autoantibodies that were thought to be anti-VGKC were found in LE patients [113,140,141]. The autoantibodies were actually targeting proteins that were in complex with the ion channel. In a following study, LGI1 was identified as the real antigen in VGKC positive patients by CBA. CASPR2 which forms part of the scaffold required to anchor the VGKC [142] was also described as an antigen [93,143].

Other neuronal surface antigens have been identified in LE patients such as autoantibodies to AMPA-R with concomitant psychotic symptoms and good response to immunotherapy [124]. The main epitopes are in the AMPA-R subunits GluR2 (6/12) followed by the GluR1 (3/10) and a GluR1/GluR2 conformational epitope (1/10) [124]. No autoantibodies against the GluR3 subunit were found. The autoantibodies bound in 91% of the cases to GluR2 in cluster with GluR3, produced a reduction in the number of GluR2 subunit in the AMPA-R clusters at the synapsis. GABAB-R autoantibodies have been recently related with an aggressive course of autoimmune encephalitis in a young patient [125]. In contrast, GABAA-R autoantibodies have been found in severe forms of encephalitis [125] e.g., in combination with anti-LGI1 in a patient presenting a subacute onset of memory loss, confabulation, and behavioral changes [126]. The D2DR has been first identified in 12 out of 17 basal ganglia encephalitis patients, an autoimmune disorder characterized by movement and psychiatric symptoms. In this study, 3 out of 12 IgG positive patients presented paranoia, psychosis and hallucinations. These autoantibodies have also been described in other neuropsychiatric disorders [129].

## 9. Psychotic Disorders

Psychotic disorders are difficult to conceptualize and thus many misconceptions still exist on what psychosis and schizophrenia are. These disorders share common symptoms that can be divided into five main categories: (i) psychosis (encompassing delusions and hallucinations—also called the positive-symptom dimension); (ii) alterations in drive and volition (the negative-symptom dimension); (iii) alterations in neuro-cognition (cognitive-symptom dimension); and (iv and v) affective dysregulation (giving rise to depressive and manic (bipolar) symptoms) [11]. The different diagnoses will be dependent on the duration and intensity of these different symptoms. Schizophrenia is the most common diagnosis within the psychotic disorders and applies to a syndrome characterized by long duration, bizarre delusions, negative symptoms, and few affective symptoms (non-affective psychosis). Other diagnoses of psychotic depression or bipolar disorder (affective psychosis) represent patients who present with a psychotic disorder with fewer negative symptoms, but with higher levels of affective (depression and mania) symptoms previous to psychosis.

The immunological involvement in psychotic disorders was already hypothesized in 1930, when some immunological signs where detected in patients with schizophrenia [156]. In Table 3, the studies involving autoantibodies in psychiatric disorders are described in detail, including an overview of the specific role of the neuronal antigens in these pathologies.

As it has been already described, NMDA-R encephalitis presents usually with an early psychotic phase and subsequent seizures, movement disorders and autonomous dysfunction, but about 4% of patients develop only isolated psychotic episodes [8,157]. NMDA-R autoantibodies have been detected in schizophrenia [122] and in some case reports, like a patient with pure typical psychotic syndrome who recovers after immunotherapy [145] and patients with a first psychotic episode post-partum [119]. Some children also show isolated psychiatric symptoms, such as a case of a 9 year-old individual diagnosed with early schizophrenia, who presented high autoantibody titers in CSF compared with serum and responded well to immunosuppressive therapy [118]. These cases are not typical because, other than in adult cases of NMDA-R encephalitis, symptoms in early life are mainly neurologic rather than psychiatric [158].

After the antigenic epitope was defined, the immunoglobulin isotype frequency was studied in a schizophrenia cohort [159]. IgGs against the GluN1a subunit were described in two first episode catatonic schizophrenic patients (probably misdiagnosed NMDA-R encephalitis), while two other paranoid schizophrenia patients presented IgGs against GluN1a/2b, in lower titers, which declined during remission also shown in other studies [115,141,145]. Curiously, only the two patients with anti-GluN1 IgG autoantibodies presented IgG positive titers in CSF, a controversial result based on the 3.2% reactivity to neuronal surface antigens in CSF found in a psychotic disorder cohort (0.8% to NMDA-R and 2.4% to VGKC complex) [144]. Another study showed GluN1 IgG autoantibodies in 5 out of 43 children with a first episode of acute psychosis, screened by a more objective variety of the CBA using flow cytometry [160]. This subunit was also recognized by IgA or IgM [160] but not specifically related with schizophrenia, since they were also present in other pathologies and in control individuals [121,159]. IgA autoantibodies to NMDA-R (but not IgG) were described in a cognitive dysfunction cohort where they are thought to induce decreased NMDA-R expression and NMDA-R mediated currents in neuronal cell cultures [120].

The frequency of autoantibodies to NMDA-R and VGKC complex in different studies ranged from 0% to 10% in cohorts of first-episode psychosis or schizophrenia [38,122,144,159,161,162]. Nevertheless, the results are controversial, since a number of other studies did not find autoantibodies in neuropsychiatric cohorts [146,159,162,163] or not specific to the disease [45,122]. New and more objective techniques, like flow cytometry CBA, are being introduced to the research routine [129,160], but a standardized procedure to screen patients for neuronal surface antigens needs to be defined to reduce results variability.

No serum autoantibodies against GluR1/GluR2 subunits from the AMPA-R were detected in a schizophrenia cohort (also not in other neuropsychiatric disorders and controls) [159]. On the other hand, the presence of autoantibodies to any class of the GABA-R has not been studied in psychotic disorders to our knowledge. The D2DR autoantibodies have been described in a cohort of first episode acute psychosis children (3 out of 43 IgG and 1 out of 43 IgM subtypes) [160].

Autoantibodies against muscarinic AChR (mAChR), specifically against the mAChR in cerebral cortex, have been identified in a small percentage of schizophrenia patients [147,164]. Similarly, autoantibodies against the α7 adult subunit of the nicotinic AChR (nAChR) were found in five out of 21 (23%) schizophrenia patients [149].

Autoantibodies to GAD have been studied in a schizophrenia cohort where no autoantibodies were found in any of the 180 CSF samples [144]. Only a single case has been reported, where a schizophrenia patient presented elevated serum titers against GAD [154].

Psychiatric manifestations consist of a broad spectrum of symptoms that can occur during the course of different disorders that do not only include the classical psychiatric disorders. We expect that some patients diagnosed with psychotic disorders like schizophrenia will in the future fall under the diagnosis of autoimmune encephalitis, yet in most cases the causative role of these autoantibodies remains to be proven. Due to the heterogeneous phenotype of mental disorders, it is important to maintain diagnostic guidelines as homogeneous, standardized and as international as possible.

## 10. Major Depressive Disorders (MDD)

Depression is a major cause of morbidity worldwide [14]. The prevalence is up to 15% of the population in industrialized nations and by 2030 it is projected by the World Health Organization to be the leading cause of disease burden globally [165]. MDD is characterized by a state of low mood and anhedonia, affecting the person's thoughts, behavior, feelings and sense of well-being [166,167]. Patients may also present with diverse symptoms including lethargy, insomnia, social withdrawal and sexual dysfunction among a range of others.

In MDD variations in ion channel and neurotransmitter receptor function are associated to the risk to develop the disorder. Serotonergic neurotransmission plays an important role in the etiology of depression [168]. The serotonin transporter (SERT) is the only known high affinity transporter which primary regulates 5HT levels in the brain and is considered a key target for widely used antidepressant drugs [17]. So alterations in SERT levels have been implicated in behavioral and neuropsychiatric disorders including MDD [17,18,169]. Genetic studies also support that polymorphisms within genes that encode for receptors or proteins involved in the serotonergic and dopaminergic systems including SERT, 1A serotonin receptor 5HT-1A, dopamine transporter (DAT) and D4 DR are associated to the risk of MDD [19].

Autoantibodies targeting neurotransmitter receptors or other neuronal antigens have been reported in association with MDD [15,16]. Roy *et al.* were the first to report high reactivity of anti-opioid receptor (OPRM1) IgG autoantibodies in 3 out of 27 patients with MDD [170]. In a later study by the same group, patient serum IgGs were isolated by affinity chromatography and analyzed for reactivity on rat brain tissue [150]. The results suggested that autoantibodies to neuronal receptors might contribute to psychiatric impairment. Susumu *et al.* replicated this study examining the presence of autoantibodies against not only OPRM1, but also 5HT-1A or 5-hydroxytryptamine receptor 1A (HTR-1A), DRD2 as well as muscarinic cholinergic receptor 1 (CHRM1) [148]. Serum IgG from patients suffering from a range of neuropsychiatric disorders, including mood disorders, was analyzed by RIA. Autoantibodies against CHRM1 in particular were significantly higher in neuropsychiatric patients than in healthy controls. However, there was no significant difference between different neuropsychiatric disorders nor any obvious correlation was found between the titer of the antibody and psychiatric symptoms [148]. The data on autoantibodies in MDD may suggest autoimmune abnormalities within the brain, however it is still unclear if they play a pathological role or they are merely bystander. Anti-NMDA-R antibodies have also been found in patients with MDD [110,171]. In contrast to anti-GluN1 autoantibodies present in autoimmune encephalitis, Larissa *et al.* proposed that anti-GluN2a antibodies are associated to depressive mood in SLE patients [171]. As described before, anti-GluN2 autoantibodies are thought to be a subset of anti-dsDNA antibodies, which could cause apoptosis of neurons *in vivo* and *in vitro* [110,172].

## 11. Autism Spectrum Disorder (ASD)

At the beginning of the 20th century Dr. Asperger described the term “autistic”, referring to patients who from the beginning of their lives have difficulties to establish human affective and interpersonal relationships [20]. Nowadays, the last edition of Diagnostic and Statistical Manual of Mental Disorders (DSM-V) includes four separate disorders under the term Autism Spectrum disorder (ASD): autistic disorder, Asperger’s disorder, childhood disintegrative disorder and the catch-all diagnosis of pervasive developmental disorder not otherwise specified. The symptomatology described in these disorders is characterized by social communication deficit and restricted interest/repetitive behaviors with high sensitivity to changes in their environment, which can be developed in different degrees of severity.

Autoimmune diseases such as rheumatoid arthritis, SLE and type 1 diabetes are strongly associated with ASD family members and ASD patients [173,174]. In a subgroup of ASD patients’ maternal autoantibody transfer may play a role in the disease. The autoantibodies are targeting the GluN2 subunit, which are enriched in female fetuses and make them more vulnerable and severely affected in fetal state than their male siblings [175]. The infusion of serum or IgGs from mothers with ASD children in pregnant mice [176,177,178] or non-human primates [68,179] reproduced the ASD-like pathology. Subsequently, neuron-reactive maternal antibodies were studied [180,181,182,183] and a pair of 37/73 and 39/73 kDa of fetal brain proteins have been specifically described as maternal antibody targets in mothers with ASD children [180,181]. In contrast, autoantibodies targeting these antigens were not detected in ASD patients [184]. Recently, seven proteins expressed in the developing brain, which are not neuronal receptors but are intracellular, extracellular and/or secreted, have been identified as fetal brain targets and they are currently used as biomarkers to predict ASD risk [185].

As discussed above, most ASD related autoantibodies that are known are transferred from the mother, yet some studies have also identified autoantibodies produced by ASD patients. These are anti-5HT receptor IgG autoantibodies [151,186], and also autoantibodies targeting non-identified antigens in the basal ganglia, prefrontal and temporal cortex, cingulate gyrus and cerebellum [187,188] specifically against the Purkinje cells [155,189]. IgGs and IgMs against brain endothelial cells were increased in ASD patients measured by (enzyme-linked immunosorbent assay) ELISA [190]. These findings were confirmed in another study by reactivity on brain endothelial cells where IgG autoantibodies were increased in ASD patients (50%) whereas none of the healthy children showed positive autoantibody staining and children without neurological illnesses to a lower degree (2 out of 21) [191]. A 45/62 kDa cerebellar protein was identified as a possible autoantigen by Western blot using rhesus macaque cerebellum homogenate. The presence of the autoantibodies correlated with a lower adaptive and cognitive function and aberrant behavior in children [192]. A 52 kDa protein located in cerebellar Golgi cells was identified in 21% of the ASD patients analyzed [193]. Later on, the same sera cohort was studied by IHC using the rostro-caudal extent of macaque brain. A specific subgroup of GABAergic interneurons located in the V1 layer was identified as the specific target [194]. Controversially, another study suggested that this staining might be unspecific because immunoreactivity was detected using serum of both healthy controls and a ASD patient with the intriguing fact that the IgG seropositive ASD patients presented more severe behavior and emotional problems compared to the IgG seronegative ones [195].

Folate receptor autoantibodies have been detected by RIA in 75.3% of ASD patients [152]. A treatment with folic acid (leucovorin) has been shown to significantly improve the ASD symptoms in at least 1 out of 3 of the individuals with ASD. The role of parental autoantibodies remains unclear, since the presence of FR autoantibodies in ASD children is not always related to the presence of the autoantibodies in the parents [196].

## 12. Obsessive-Compulsive Disorder (OCD) and Attention-Deficit/Hyperactivity Disorder (ADHD)

The mental disorders explained below are two of the most common neuropsychiatric diseases in early life patients. OCD patients develop pathological hoarding behaviors characterized by obsessions (recurrent intrusive thought) and/or compulsions or tics (repetitive or serotyped behaviors) like recurrent skin-piking resulting in skin lesions. ADHD is the most prevalent chronic neurodevelopmental disorder in school age children, affecting 5%–8% [197,198] and being more frequent in boys than in girls. In two thirds of these cases, the disease coexists with other conditions like tics or the Tourette syndrome. It is characterized by hyperactivity, impulsiveness and long lasting inattention.

The relationship between post-streptococcal infections immunity and OCD and ADHD has been widely studied. Obsessions and compulsions were observed in post-streptococcal infection and pediatric autoimmune neuropsychiatric disorders, then commonly referred to as pediatric autoimmune neuropsychiatric disorders associated with streptococcal infections (PANDAS) [199,200,201]. An animal model was established to study if these antibodies cause neuropsychiatric symptoms [202]. Plasma exchange affects the disease and removal of the autoantibodies could cause improvement of symptoms in OCD and tic disorders in childhood [203]. In recent years, more studies revealed that anti-basal ganglia antibodies (ABGA) actually could target neuronal surface antigens. Kirvan *et al.* found that autoantibodies in PANDAS could bind to neuronal surface and caudate-putamen, which could activate CaM kinase II and cause behavioral disorders [83,204]. Brimberg *et al* first reported that autoantibodies could bind to DRD1 and DRD2 after the immunization with streptococcal antigen which leads to neuropsychiatric symptoms in the rat animal model [205]. Lately, Lotan and colleagues reported that rats exposed to group A streptococcal antigens developed compulsive-like behavior. Serum IgG from group A streptococcal-exposed rats reacted with DRD1 and DRD2 and 5HT-2A and 5HT-2C serotonin receptors *in vitro* (determined by ELISA and Western blot). *In vivo*, IgG deposits in the striatum of infused rats colocalized with specific brain proteins such as DR and SERT (by IHC), suggesting that the autoantibodies are the cause of the compulsive-like and motor dysfunction behavior observed in the animals [206]. Autoimmunity is, arising from an abnormal immune response, probably due to the high mimicry found between pathogens and neuronal surface epitopes involved in the dopaminergic and the serotonergic system. These findings link post infectious autoimmunity to the onset of both OCD and ADHD. Giana *et al.* found elevated IgG autoantibody titers against DAT in the serum of ADHD children by ELISA (*n* = 61) [153], which again suggests that the dysregulation in the levels of dopamine neurotransmitter may be caused by autoantibodies in these disorders.

## 13. Conclusions/Future Directions

NMDA-R autoantibodies can cause neuropsychiatric symptoms which can range from purely psychiatric to encephalitis with neurological symptoms. Due to the overlap in symptoms with psychotic disorders, some patients might be misdiagnosed as first episode psychosis or even schizophrenia. We see the need for a systematic screening of neuronal autoantibodies in psychotic patients, especially in the early phase of the disease to improve diagnosis of autoimmune psychotic disorders. Preferably, hereby not only serum, but also CSF would be screened because antibody titers might be low in the blood and only detectable in CSF (e.g. due to intrathecal antibody synthesis). The diagnosis of autoimmune neuropsychiatric diseases could be challenging for many clinicians and the acceptance and implementation of these diagnostic procedures differs widely between countries. The communication between the disciplines of neurology and psychiatry is of high importance in these cases, because not only antibody testing but also neurological testing might improve the diagnosis of these patients. Mild neurological symptoms are frequent in patients with psychotic symptoms [207] but could be missed because they are (a) difficult to examine in the presence of acute psychiatric symptoms; (b) less attention is paid on these examinations and (c) neurological symptoms are attributed to side effect of psychopharmacological therapy. Psychiatrists thus need to be aware of this new diagnosis in a subset of psychotic patients and education has to be provided to also perform neurological examinations or form a multidisciplinary team with neurologists (also for CSF sampling).

Still, the prevalence of these autoantibodies in cohorts of schizophrenia, bipolar disorder or other mental illnesses is not determined due to high variation in the current research results. The variable results in the field might be caused by small sample sizes, the heterogeneity in patient cohorts, the unclear distinction between the different mental disorders (especially in psychotic disorders), stage of disease and also by methodological differences. The most commonly used diagnostic methods are the CBA and IHC on rat brain. Nevertheless, these methods are still relatively new with room for improvement. Adequate training is necessary to interpret the results and to avoid false conclusions as they have been reported before [163,208]. The IHC is limited by high background with serum stainings. The other neuronal autoantibodies described here are still lacking final proof to confirm that they are causing a neuropsychiatric autoimmune disease. Indications for autoimmunity are that several of these autoantibodies targeting VGKC complex, mAChR and DRD2 receptor are present with the clinical manifestation of psychotic disorders including MDD, that they are detectable in the blood and CSF and also that they target a receptor, ion channel, or other protein expressed on the cell surface which is related to symptoms. To confirm the pathogenic role of isolated autoantibodies according to Witebsky’s postulates, (monoclonal) antibody transfer should be shown to replicate the disease in an animal experimental model or in humans (maternal transfer) and that elimination or suppression of the autoimmune response by therapy can prevent disease progression or reduces the clinical manifestations. To this end, larger systematic, multicenter clinical studies are necessary to reveal the prevalence of different neuronal autoantibodies in neuropsychiatric disorders and whether these patients react to immunotherapy. Additionally, it is especially important to not only analyze sera but also CSF (see above) [116]. Table 4 highlights the relevance of the discussed autoantibodies in neuropsychiatric diseases and summarizes what remains unknown. Presumably, a number of antigens involved in neuropsychiatric disorders are still not well understood demanding further work to identify novel autoantibody targets and help to better diagnose autoimmune patients’ subgroups and understand disease mechanisms.

### To take home…

- Neuronal surface autoantibodies cause neuropsychiatric symptoms in a subgroup of the patients.
- Antibody screening and neurological examinations should be implemented to improve diagnosis of autoimmune psychotic disorders.
- Limited sample sizes, differences in patient cohorts and stage of the disease but also methodological differences generate high variation in current results.
- Common techniques, more sensitive and reproducible, are required to standardize the diagnostic tools for the different neuronal antigens across-laboratories.
- It is important to implement CSF analysis in neuropsychiatric disorder diagnosis routine, since some autoantibodies are only detectable in CSF.
- More animal studies are needed to unravel the pathogenic effect of the autoantibodies in the CNS.

## Figures and Tables

**Table 1:** Description of the characteristics, prevalence and etiology of the mental disorders.

Disorder	Characteristics	Prevalence	Etiology
Psychotic disorders	Delusions, hallucinations, disorganized speech and behavior, and other symptoms. Social or occupational dysfunction.	Estimates of the prevalence vary greatly. The median European prevalence is ~5.3%, with interquartile range of 1.9%–14.4% [9].	Environmental and genetic factors; about 80% of heritability [10,11,12,13].
Major depressive disorder (MDD)	Feelings of persistent sadness and anhedonia that affect thoughts and behavior. Leading to physical problems. Major cause of morbidity worldwide [14].	Prevalence is up to 15% of the population.	Environmental and genetic factors; possibly autoantibody involvement [15,16,17,18,19].
Autism spectrum disorder (ASD)	Social communication deficit, restricted interest, repetitive behaviors with high sensitivity to changes in environment. Difficulty to establish human affective and interpersonal relationships [20].	Prevalence of 1.47% in 2010 [21], increased over the time, males being 5 times more affected than females [22].	Environmental and genetic factors; ~90% heritability [23].
Attention-deficit/hyperactivity disorder (ADHD)	Inattention, hyperactivity and impulsivity like excessive talking, fidgeting, or an inability to remain seated in appropriate situations. Incapability to focus and organize tasks and activities.	Most prevalent chronic neurodevelopmental disorder in school age children, affecting 2-18% [24,25] and being more frequent in males than in females.	Strong genetic link as well as environmental factors [25]; heritability ~76% [26]; post-infectious autoimmunity [27].
Obsessive-Compulsive disorder (OCD)	Anxiety, recurrent unwanted thoughts (obsessions) and repetitive behaviors (compulsions).	Affects 1%–3% of the worldwide population [28,29,30].	Genetic and environmental factors [31]; heritability of ~50% in children [32] post-infectious autoimmunity [33,34].

**Table 2:** Autoantibodies in neurologic diseases with psychiatric symptoms.

Antigen Target	Subunit/Associated Protein	Related Disease	n+/n Patient	n+/n Control	Age Range *	Ig Type	Ref.
Autoantibodies to neuronal surface antigens **							
VGKC complex	n.s. ***	Limbic encephalitis	4/15	n.t. ***	47–69	IgG	[113]
NMDA-R	GluN1	NMDA-R encephalitis with psychiatric symptoms	50/485	n.t	17–44	IgG	[114]
			100/100	n.t	5–76	IgG	[115]
			250/250	0/100	n/a	IgG	[116]
			6/505	n.t	18–35	IgG	[117]
		NMDA-R encephalitis (isolated psychiatric episodes)	571/571	n.t	12–62	IgG	[8]
		NMDA-R encephalitis (schizophrenia, and autism)	1/1	n.t	9	IgG	[118]
		Autoimmune encephalitis in postpartum psychosis	2/96	0/64	25, 31	IgG	[119]
	GluN2a/2b	Progressive cognitive dysfunction of unclear etiology	7/24	n.t	49–81	IgA	[120]
		Herpes simplex encephalitis	5/44 9/44 9/44	n.t	24–79	IgG IgM IgA	[121]
		Limbic encephalitis, narcolepsy	3/5, 3/5	n.t	18–59, 24–61	IgG	[122]
	GluN1/GluN2a/2b	NMDA-R encephalitis associate with ovarian teratoma	12/12	0/200	14–44	IgG	[123]
AMPA-R	GluA1, GluA2	Limbic encephalitis	22/62	n.t	23–81	n/a	[124]
GABA-R	Type B	Encephalitis with opsoclonus, Ataxia, Chorea and Seizures	1/1	n.t	3	IgG	[125]
	α1/β3 subunits	Encephalitis with refractory seizures, status epilepticus,	6/140	0/75	n/a	IgG	[125]
	α1/β3 subunits	Encephalitis with thymoma	1/1	n.t	45	IgG	[126]
GlyR	α1	Progressive encephalomyelitis with rigidity and myoclonus (PERM)	52/779	n.t	1–75	IgG	[102]
mGluR	mGluR5	Encephalitis (Hodgkin lymphoma, Ophelia syndrome)	2/2	n.t	15, 46	IgG	[127]
Kv4.2	DPPX	Encephalitis (subacute onset of neuropsychiatric symptoms)	4/4	0/210	45–76	IgG	[128]
D2DR	D2	Basal ganglia encephalitis **, Sydenham’s chorea **, Tourette’s syndrome **	12/17, 10/30, 4/44	0/67	1–15, 2–17, 2–13	IgG	[129]
Folate receptor	-	Cerebral folate deficiency syndrome	25/28	0/28	2.5–19.3	n/a	[130]
Autoantibodies to (neuronal) intracellular antigens **							
Rib-P	P1, P2, P3	SLE with Depression	22/100	n.t	23–36	IgG	[105]
GAD	n.s.	Non-paraneoplastic limbic encephalitis	2/2	n.t	20,47	IgG	[131]

* Age range of the positive subjects; ** Anti-basal ganglia antibodies (ABGA) can bind to either neuronal surface or intracellular antigens and are related to basal ganglia encephalitis, Sydenham’s chorea, Tourette’s syndrome, OCD and ADHD. For details see OCD and ADHD sections; *** n.s. = not specified; .n.t. = not tested; VGKC complex = voltage gated potassium channel complex; NMDA-R: *N*-Methyl-d-Asparte receptor; AMPA-R = α-amino-3-hydroxy-5-methyl-4-isoxazolepropionic acid receptor; GABA-R = γ-aminobutyric acid; GlyR = glycine receptor; mGluR= metabotropic glutamate receptor; Kv4.2 = Potassium channel, voltage dependent, Kv4.2; DPPX = Dipeptidyl-Peptidase-Like Protein-6; DRD2 = dopamine-2 receptor; Rib-P = ribosome P protein; SLE = Systemic lupus erythematosus; GAD = glutamic acid decarboxylase.

**Table 3:** Autoantibodies related to psychiatric disorders.

Antigen Target	Subunit/Associated Protein	Related Disorders (D)	n+/n Patient	n+/n Control	Ig Type	Age Range *	Ref.
Autoantibodies to neuronal surface antigens **							
VGKC complex	LGI1, CASPR2	Psychotic D	3/125	n/t ***	IgG	n/a ***	[144]
	n.s. ***	Psychotic D (schizophrenia)	1/46	n/t	IgG	22 (pp)	[145]
NMDA-R	GluN1	Psychotic D and major depressive D. (n.s.)	81/1688	74/1703	IgM	26–56 (p)	[146]
			92/1688	76/1703	IgA		
			14/1688	20/1703	IgG		
	GluN2a/2b	Psychotic D (schizophrenia)	4/51	n/t	IgG	26–53 (pp)	[122]
		Psychotic D (schizophrenia)	3/46	n/t	IgG	19–28 (pp)	[145]
Muscarinic AChR	M1,M2	Schizophrenia	(n/a)/21	(n/a)/25	IgG	25–56 (p)	[147]
		Psychotic D, bipolar and depressive D	42/122	0/52	n/a	24–63 (p)	[148]
Nicotinic AChR	α7	Schizophrenia	5/21	0/17	IgG	46–61 (pp)	[149]
D2DR	D2	Bipolar and major depressive D	6/122	0/52	n/a	30–63 (p)	[148]
Opioid receptor	OPRM1	Psychotic D, bipolar and major depressive D	16/122	0/52	n/a	30–63 (p)	[148]
		Major depressive D	2/27	n/a	IgG	n/a	[150]
5HT receptor	HTR1A	Psychotic D, Major depressive D	9/63	0/52	n/a	30–63 (p)	[148]
		Autism spectrum D (Autism)	n/a	n/a	n/a	<10 (pp)	[151]
FR	-	Autism spectrum D (Autism)	70/93	n/t	n/a	3–18 (t)	[152]
DAT	-	Attention-deficit/hyperactivity	n/a/46	n/a/15	IgG	4–16 (t)	[153]
Autoantibodies to (neuronal) intracellular antigens **							
GAD	GAD 65	Psychotic D (Schizophrenia)	1/1	n/t	n/a	19 (pp)	[154]
		Autism spectrum D (Autism), Attention-deficit/hyperactivity D	3/20, 4/15	0/14	IgG	8–11 (pp)	[155]

* Age range: (pp) the age range belongs to the patients tested positive in the assay, (p) the age range belongs to the total patients cohort tested in the assay, (t) the age range belongs to all the subjects (including controls and patients) tested in the assay; ** Anti-basal ganglia antibodies (ABGA) can bind to either neuronal surface or intracellular antigens and are related to OCD, ADHD. For details see paragraph on OCD and ADHD; *** n.s. = not specified; n/a = not available. n/t = not tested; VGKC complex = voltage gated potassium channel complex; Ig = immunoglobulin G; NMDA-R: *N*-Methyl-d-Asparte receptor; AChR = acetylcholine receptor; DRD2 = dopamine-2 receptor; OPRM1 = opioid receptor, mu 1; 5H = serotonin; HTR1A = 5-Hydroxytryptamine (Serotonin) Receptor 1A; FR = Folate receptor; DAT = Dopamine transporter; GAD= glutamic acid decarboxylase.

**Table 4:** Evidence of autoantibody-mediated mechanisms in neuropsychiatric disorders.

Disorders	Targets of the Autoantibodies	Prevalence *		*in Vitro* *		*in Vivo* *		Immunotherapy *	
**Psychotic**	NMDA	+/−	[122,145,146]	+	[86,114,120]	+	[137]	+	[145]
	VGKC complex	+/−	[144,145]	+	[93]	n/a **		+	[145]
	AMPA-R	−	[144,159]	+	[209,210]	+	[211]	n/a	
	D2DR	+	[148,160]	+	[129]	n/a		n/a	
	HTR-1A	+	[148]	n/a		n/a		n/a	
	mAChR	+	[147,148]	+	[147,164]	n/a		n/a	
	nAChR	+	[149]	n/a		n/a		n/a	
	GAD	+/−	[144,154]	−	[99]	n/a		+	[154]
	FR	+	[202,212]	n/a		n/a		n/a	
**Major depressive**	OPRM1	+	[148,150]	n/a		n/a		n/a	
	D2DR	+	[148]	n/a		n/a		n/a	
	HTR-1A	+	[148]	n/a		n/a		n/a	
	mAChR	+	[148]	n/a		n/a		n/a	
	NMDA-R	+	[146]	+	[86]	+	[137]	n/a	
	Rib-p	n/a		+	[213]	+	[106]	n/a	
**Autism**	HTR-1A	+	[151]	n/a		n/a		n/a	
	FR	+	[152]	n/a		n/a		n/a	
	GAD	+	[155]	n/a		n/a		n/a	
**Obsessive-compulsive**	Basal ganglia	+/−	[34,214,215]	+	[204]	+	[202,206]	+	[203]
	D2DR	n/a		+	[216]	+	[205]	n/a	
**Attention deficit hyperactivity**	Basal ganglia	+/−	[217,218]	+	[204]	n/a		n/a	
	GAD	+/−	[155,219]	n/a		n/a		n/a	

* All autoantibodies mentioned above have been reported in biological fluids in human subjects with neuropsychiatric disorders. Data shown here does not include autoimmune encephalitis. The pathological role of the autoantibodies has not been demonstrated for all cases; Coding is done as follows: Column for prevalence (Pre): Antibody is more frequent in the specific patients’ cohorts than in healthy individuals (+), not (−), or not available (n/a); *in vitro*: Autoantibody shows toxicity to cells *in vitro* or could change the antigen function (+), not (−), or not available (n/a); *in vivo*: Animal studies show the autoantibody could cause neuropsychiatric behavior (+), not (−), or not available (n/a); Immunotherapy: Patients would benefit from immunotherapy (+); if immunotherapy was not beneficial (−), if data not available (n/a); ** n/a = not available; NMDA-R = *N*-Methyl-d-Asparte receptor; VGKC complex = voltage gated potassium channel complex; AMPA-R = α-amino-3-hydroxy-5-methyl-4-isoxazolepropionic acid receptor; DRD2 = dopamine-2 receptor; HTR1A = 5-Hydroxytryptamine (Serotonin) Receptor 1A; AChR = acetylcholine receptor; GAD = glutamic acid decarboxylase; FR = Folate receptor; OPRM1= opioid receptor, mu 1.

## Abbreviations

The following abbreviations are used in this manuscript:

5HT	serotonin
5HT-R	serotonin receptor
ABGA	Anti-basal ganglia antibodies
ACh	acetylcholine
AChR	acetylcholine receptor
AChRγ	gamma subunit of the peripheral neuronal ACh receptor
ADHD	attention-deficit/hyperactivity disorder
AMPA-R	α-amino-3-hydroxy-5-methyl-4-isoxazolepropionic acid receptor
ApoE	apolipoprotein E
ASD	Autism Spectrum disorder
BBB	blood-brain barrier
BD	bipolar disorder
CASPR2	contactin associated protein-2
CBA	cell based assay
CHRM1	muscarinic cholinergic receptor 1
CNS	central nervous system
CSF	cerebrospinal fluid
D	Disorder
DAT	dopamine transporter
DPPX	Dipeptidyl-Peptidase-Like Protein-6
DRD2	dopamine-2 receptor
dsDNA	double stranded DNA
FcRn	neonatal Fc receptors
FR	folate receptor
GABA	γ-aminobutyric acid
GABAA-R	A class of the GABA-R
GABAB-R	B class of the GABA-R
GABA-R	γ-aminobutyric acid receptor
GAD	glutamic acid decarboxylase
Glu	glutamate
Gly	glycine
GlyR	glycine receptor
HLA	human leukocyte antigen
HTR-1A	5-hydroxytryptamine receptor 1A
IgG	immunoglobulin G
IHC	immunohistochemistry
LE	limbic encephalitis
LGI1	leucine-rich glioma inactivated-1
mAChR	muscarinic AChR
MDD	major depressive disorder
MG	myasthenia gravis
mGluR	metabotropic glutamate receptor
MHC	major histocompatibility complex
nAChR	nicotinic AChR
NMDA-R	*N*-Methyl-d-Asparte receptor
NMJ	neuromuscular junction
OCD	Obsessive-Compulsive disorder
PNS	peripheral nervous system
RIA	radioimmunoassay
Rib-P	ribosome P protein
SERT	serotonin transporter
SC	Sydenham chorea
SLE	systemic lupus erythematous
VGKC complex	voltage gated potassium channel complex
