# Addendum: Hoffmann, C.; et al. Autoantibodies in Neuropsychiatric Disorders. *Antibodies* 2016, *5*, 9

**DOI:** 10.3390/antib7030033

**Published:** 2018-08-27

**Authors:** Carolin Hoffmann, Shenghua Zong, Marina Mané-Damas, Peter Molenaar, Mario Losen, Pilar Martinez-Martinez, Antibodies Editorial Office

**Affiliations:** 1 Division Neurosciences, School for Mental Health and Neurosciences, Maastricht University, 6200 MD Maastricht, The Netherlands; c.hoffmann@maastrichtuniversity.nl (C.H.); s.zong@maastrichtuniversity.nl (S.Z.); m.damas@maastrichtuniversity.nl (M.M.-D.); p.molenaar@maastrichtuniversity.nl (P.M.); m.losen@maastrichtuniversity.nl (M.L.); p.martinez@maastrichtuniversity.nl (P.M.-M.); 2 MDPI, St. Alban-Anlage 66, 4052 Basel, Switzerland

The conflict of interest section was missing in the published paper [1]. It has been updated as follows:

“The authors declare no conflict of interest.”

The manuscript will be updated and the original will remain online on the article webpage.
